# Supplementary material for: Association between Symptoms of Depression and Generalised Anxiety Disorder Evaluated through PHQ-9 and GAD-7 and Anti-Obesity Treatment in Polish Adult Women
Source: Nutrients. 2024 Jul 26;16(15):2438. doi: 10.3390/nu16152438 (PMC11314622; doi:10.3390/nu16152438)
Supplement: Supplementary file 1 [file nutrients-16-02438-s001.zip › nutrients-3086354-supplementary.pdf]

Supplementary Material Table S1.

The English Version of the Study Questionnaire

**Demographic variables**	
How old are you? [years]	
Sex	Female
	Male
	Other
What is your height? [in centimeters]	
What is your weight? [in kilograms]	
Is this currently your highest body weight in your life? For women, if applicable, please exclude weight from pregnancy and postpartum period (6 weeks after childbirth).	Yes
	No
Additional question after choosing „No” in Q: Is this currently your highest body weight in your life?	
What was your highest body weight in your life? [in kilograms]	
Are you currently using any of the listed medications for treating obesity? (multiple choice)	Saxenda or Victoza (active ingredient liraglutide)
	Ozempic or Wegovy (active ingredient semaglutide)
	Mysimba (active ingredient Bupropion Hydrochloride + Naltrexone Hydrochloride)
	I don't use any of the above
Have you undergone bariatric surgery in the past?	Yes
	No
Additional question after choosing „Yes” in Q: Have you undergone bariatric surgery in the past?	
What kind of procedure did you have?	Gastric Sleeve surgery

	Gastric Bypass surgery
	Gastric Band surgery
	Endoscopic intragastric balloon
	Other
Do you have any chronic illnesses?	Yes
	No
Additional question after choosing „Yes” in Q: Do you have any chronic illnesses?	
What chronic illness do you have? (multiple choice)	Obesity
	Hypertension
	Cardiovascular disease other than hypertension
	Diabetes Mellitus type 2
	Arthritis
	Hypothyroidism
	Depressive Disorder
	General Anxiety Disorder
	Dyslipidemia
	Hepatic Steatosis
	Other, not mentioned above
**TFEQ-R18 Questionnaire**	
I deliberately take small helpings as a means of controlling my weight.	Definitely true
	Mostly true

	Mostly False
	Definitely False
When I feel anxious, I find myself eating.	Definitely true
	Mostly true
	Mostly False
	Definitely False
Being with someone who is eating often makes me hungry enough to eat also.	Definitely true
	Mostly true
	Mostly False
	Definitely False
When I feel anxious, I find myself eating.	Definitely true
	Mostly true
	Mostly False
	Definitely False
When I see a real delicacy, I often get so hungry that I have to eat right away.	Definitely true
	Mostly true
	Mostly False
	Definitely False
I get so hungry that my stomach often seems like a bottomless pit.	Definitely true
	Mostly true
	Mostly False

	Definitely False
I am always hungry so it is hard for me to stop eating before I finish the food on my plate.	Definitely true
	Mostly true
	Mostly False
	Definitely False
When I feel lonely, I console myself by eating.	Definitely true
	Mostly true
	Mostly False
	Definitely False
I deliberately take small helpings as a means of controlling my weight.	Definitely true
	Mostly true
	Mostly False
	Definitely False
I do not eat some foods because they make me fat.	Definitely true
	Mostly true
	Mostly False
	Definitely False
I am always hungry enough to eat at any time.	Definitely true
	Mostly true
	Mostly False
	Definitely False

When I smell delicious food, I find it very difficult to keep from eating, even if I have just finished a meal.	Definitely true
	Mostly true
	Mostly False
	Definitely False
Sometimes when I start eating, I just can't seem to stop.	Definitely true
	Mostly true
	Mostly False
	Definitely False
How often do you feel hungry?	Only at meal times
	Sometimes between meals
	Often between meals
	Almost always
How likely are you to consciously eat less than you want?	Unlikely
	Slightly likely
	Moderately likely
	Very likely
How frequently do you avoid "stocking up" on tempting foods?	Almost never
	Seldom
	Moderately likely
	Almost always
Do you go on eating binges though you are not	Never

hungry?	Rarely
	Sometimes
	At least once a week
How much do you restrict your food intake? Mark on a scale from 1 to 8.	1 (never restricting)
	2
	3
	4
	5
	6
	7
	8 (always restricting)
**General Anexiety Disorder-7 Questionnaire**<br>Over the last two weeks, how often have you been bothered by the following problems?	
Feeling nervous, anxious, or on edge.	Not at all
	Several days
	More than half the days
	Nearly every day
Not being able to stop or control worrying.	Not at all
	Several days
	More than half the days
	Nearly every day
Worrying too much about different things.	Not at all

	Several days
	More than half the days
	Nearly every day
Trouble relaxing.	Not at all
	Several days
	More than half the days
	Nearly every day
Being so restless that it is hard to sit still.	Not at all
	Several days
	More than half the days
	Nearly every day
Becoming easily annoyed or irritable.	Not at all
	Several days
	More than half the days
	Nearly every day
Feeling afraid, as if something awful might happen.	Not at all
	Several days
	More than half the days
	Nearly every day
**Patients Health Questionnaire-9 (PHQ-9)**<br>Over the last 2 weeks, how often have you been bothered by any of the following problems?	
Little interest or pleasure in doing things.	Not at all

	Several days
	More than half the days
	Nearly every day
Feeling down, depressed, or hopeless.	Not at all
	Several days
	More than half the days
	Nearly every day
Trouble falling or staying asleep, or sleeping too much.	Not at all
	Several days
	More than half the days
	Nearly every day
Feeling tired or having little energy.	Not at all
	Several days
	More than half the days
	Nearly every day
Poor appetite or overeating.	Not at all
	Several days
	More than half the days
	Nearly every day
Feeling bad about yourself — or that you are a failure or have let yourself or your family down.	Not at all
	Several days

	More than half the days
	Nearly every day
Trouble concentrating on things, such as reading the newspaper or watching television.	Not at all
	Several days
	More than half the days
	Nearly every day
Moving or speaking so slowly that other people could have noticed? Or the opposite — being so fidgety or restless that you have been moving around a lot more than usual	Not at all
	Several days
	More than half the days
	Nearly every day
Thoughts that you would be better off dead or of hurting yourself in some way.	Not at all
	Several days
	More than half the days
	Nearly every day
